# Interdisciplinary and multiprofessional outpatient secondary individual prevention of work-related skin diseases in the metalworking industry: 1-year follow-up of a patient cohort

**DOI:** 10.1186/s12895-018-0080-2

**Published:** 2018-12-12

**Authors:** Annika Wilke, Günther Gediga, Andreas Goergens, Andreas Hansen, Anja Hübner, Swen Malte John, Kathrin Nordheider, Marc Rocholl, Sabine Weddeling, Britta Wulfhorst, Dorothée Nashan

**Affiliations:** 1 Institute for Health Research and Education, Department of Dermatology, Environmental Medicine and Health Theory, University of Osnabrück, Am Finkenhügel 7a, 49076 Osnabrück, Germany; 2 Institute for Interdisciplinary Dermatological Prevention and Rehabilitation (iDerm) at the University of Osnabrück, Am Finkenhügel 7a, 49076 Osnabrück, Germany; 3 German Social Accident Insurance Institution for the woodworking and metalworking industries, district administration in Dortmund, Semerteichstraße 98, 44263 Dortmund, Germany; 4 Department of Dermatology, Klinikum Dortmund gGmbH, Beurhausstr. 40, 44137 Dortmund, Germany; 5 Faculty of Human Sciences/Department of Educational Sciences, MSH Medical School Hamburg, University of Applied Sciences and Medical University, Am Kaiserkai 1, 20457 Hamburg, Germany

**Keywords:** Occupational contact dermatitis, Hand eczema, Prevention, Metalworking industry, Patient education, Occupational health, Skin protection, Patient care, Follow-up, Interdisciplinary

## Abstract

**Background:**

In Germany, work-related skin diseases are predominant within the spectrum of reported occupational diseases. Metal workers are among the high-risk professions. Offering effective prevention programs to affected patients is of utmost importance to avoid deterioration of the disease and job loss. We conducted a 1-year follow-up in patients who participated in a multidisciplinary, complex outpatient prevention program representing a standard procedure of patient care by the respective statutory accident insurance.

**Methods:**

The multi-component prevention program consists of multiprofessional individual patient counseling, a structured skin protection seminar in a group, as well as workplace visits and on-site counseling in terms of appropriate skin protection (e.g. gloves). An observational study with a 1-year follow-up and four measurements (T1-T4, longitudinal pre/post-test design) including dermatological examinations and standardized written questionnaires was conducted between 2013 and 2016 to assess changes over time regarding job loss and disease severity.

**Results:**

Data from 94 patients (87 male, mean age: 45.4 years) were included in the analysis. One year after the skin protection seminar (T4), 83 patients (88.3%) remained in their original professional metalworking activity and four patients (4.3%) had given up their profession because of their skin disease. At baseline (T1), irritant contact dermatitis of the hands was the most frequent diagnosis (80.7%). Methods for self-reported disease severity showed good correlation with the clinical gold standard at T1 and T2 (dermatological examination with the Osnabrück Hand Eczema Severity Index / OHSI), and a significant decrease of the self-reported disease severity was found over time from T1 to T4 (*p* < 0.001). Further results indicate an improved self-perceived disease control and an overall satisfaction with the prevention program.

**Conclusions:**

The results of this observational study demonstrate that the comprehensive prevention program positively influences the course of work-related skin diseases, increases the possibility to continue working in a “high-risk” profession and improves the disease management of metal workers. In the long term, the prevention program may lead to cost savings by preventing high therapy costs or professional retraining.

**Electronic supplementary material:**

The online version of this article (10.1186/s12895-018-0080-2) contains supplementary material, which is available to authorized users.

## Background

For the past decades, suspected cases of “severe or recurrent skin diseases” (occupational disease no. 5101, a disease which is “so severe as to have forced the person to discontinue all activities that caused or could cause the development, worsening or recurrence of the disease” [1]), are predominant within all work-related diseases reported annually to the German Statutory Social Accident Insurance bodies [1, 2]. Work-related skin diseases (WRSD), mainly irritant and/or allergic contact dermatitis, are of great medical and socio-economic concern because they impair the well-being and quality of life. They can provoke long periods of absenteeism due to illness and inability to work or may even require job change, which generates high direct and indirect costs [3–5].

In Germany, for dermatologists it is mandatory to immediately inform the responsible Statutory Social Accident Insurance body of any suspected WRSD as part of the so-called ‘dermatologist’s procedure’ (‘Hautarztverfahren’). In response, the Statutory Social Accident Insurance body initiates a hierarchical multi-step intervention procedure (‘Verfahren Haut’) [6–10]. Procedures and the prevention measures are adapted to the individual disease severity starting with outpatient dermatological therapy and outpatient prevention programs in case of milder forms of WRSD and increasing to inpatient rehabilitation for recalcitrant, severe WRSD [3, 6, 8, 10]. The effectiveness of outpatient and inpatient prevention programs have been shown in previous studies [3, 11–13]. Thus, these programs have been integrated in the regular patient care by most German Statutory Social Accident Insurance bodies [6, 7, 10].

In the past, several studies on the effectiveness of outpatient interdisciplinary secondary prevention have been published with a special focus on hairdressers, health care workers as well as cleaning and kitchen employees [11–18]. The prevention programs usually consist of both health educational and dermatological elements. These programs aim at a) enabling the patients to remain in their professional activity despite their skin disease, and b) positively influencing the individual disease management and skin protection behavior [11–18]. They usually focus on workers’ individual change of knowledge, attitudes and skin protection behavior (behavior-oriented approaches). However, there are occupational areas, such as metalworking professions, which require more structurally-oriented prevention approaches that systematically consider the actual and individual workplace situation due to very specific demands pertaining to skin protection.

Metal workers are well-known to be at risk of developing work-related irritant and allergic contact dermatitis (ICD/ACD) [19–23]. Apfelbacher et al. found a cumulative incidence of 29.3% in the car industry over a study period of more than 10 years [21]. Typical skin exposure in metal workers is the repetitive contact with subtoxic irritants and allergens (for instance metalworking fluids, cleaning detergents, solvents, skin cleaning procedures) [20, 22, 23]. However, under the term of metalworking professions a broad spectrum of workplace settings and associated skin hazards is included, for instance regarding the individual processing methods applied or regarding particular hazards (e.g., rotating machines and workpieces, heat and sparks, specific mechanical risks, or chemicals of different hazards and concentrations). As a consequence, since 2007 a unique interdisciplinary outpatient prevention program was developed specifically for metal workers affected by WRSD as a part of the regular patient care. Based on a cooperation between different institutions and professions it systematically combines approaches of behavior-oriented and structural prevention at workplace (e.g. on-site identification of risks and skin protection measures).

To the best of our knowledge, no follow-up data have been published until now that report on the effects of this kind of outpatient prevention program in metal workers one year after participation. Thus, this paper presents 1-year follow-up results of participants for the primary outcomes “remaining in work” and “disease severity”. Based on previous studies in other branches [11–13, 15, 17], our assumptions were that the majority of the trained patients remain in their professional metalworking activity and that the disease severity significantly improves after one year.

## Methods

### Aim, prevention approach and timeline

The prevention program examined in our study aims at enabling metal workers suffering from WRSD to remain in work without skin lesions. Since June 2007, the program is embedded in a standard procedure of patient care and case management applied by the Social Accident Insurance Institution for the woodworking and metalworking industries, district administration in Dortmund, in cooperation with the Department of Dermatology, Hospital of Dortmund and the University of Osnabrück. In this program, a suspected case of WRSD is initially reported to the Social Accident Insurance Institution for the woodworking and metalworking industries via the so-called ‘dermatologist*’*s report’ completed by a local dermatologist or an occupational physician [6–9]. If the patient agrees, he or she will be visited by an employee of the responsible Statutory Social Accident Insurance (prevention services, formerly: technical inspectorate) at the workplace within the first eight weeks after notification in order to analyze the skin exposure to workplace hazards, to identify possibilities to improve the skin protection, and to provide individual counseling. If necessary, skin protection products (e.g., gloves, creams) are recommended. If the employer and/or the health and safety officer agree(s), products are tentatively provided for free by the Statutory Social Accident Insurance. Additionally, if required, employees of the prevention services have the legal authority to demand certain changes to improve health and safety at the workplace. If needed, regular follow-ups on-site and/or by phone are conducted by the employee of the prevention services to monitor the course of the WRSD and to consider alternative prevention measures. Concomitantly, the patient is followed in continuous dermatological treatment by a local dermatologist.

Under certain conditions, the patients will be referred to a specific patient management pathway. This is the case if the employee of the prevention services a) identifies an individual need for intensified health education and counseling, b) does not observe any improvement of the WRSD, c) has the impression that preventive measures are exhausted, d) the local dermatologist reports a clinically severe form of WRSD or the need for health education and counseling.

This specific care pathway of patient management is investigated by this study. As a first step (T1), the patients are examined by a trained dermatologist at the Department of Dermatology of the Hospital of Dortmund. The department is specialized in occupational dermatology, diagnostic approaches and treatment of WRSD. The results of these dermatological consultations are compiled in a detailed report, which is sent to the Statutory Social Accident Insurance. The report includes information on the occupation, skin exposure to irritants and allergens, the course, localization and severity of the skin disease, atopy, patch and prick test results, skin protection products, and, if applicable, recommendations for the local dermatologist and the accident insurance concerning further diagnostics, therapy options, workplace visits, optimization of skin protection, and/or inpatient/outpatient prevention measures.

**Fig. 1:**
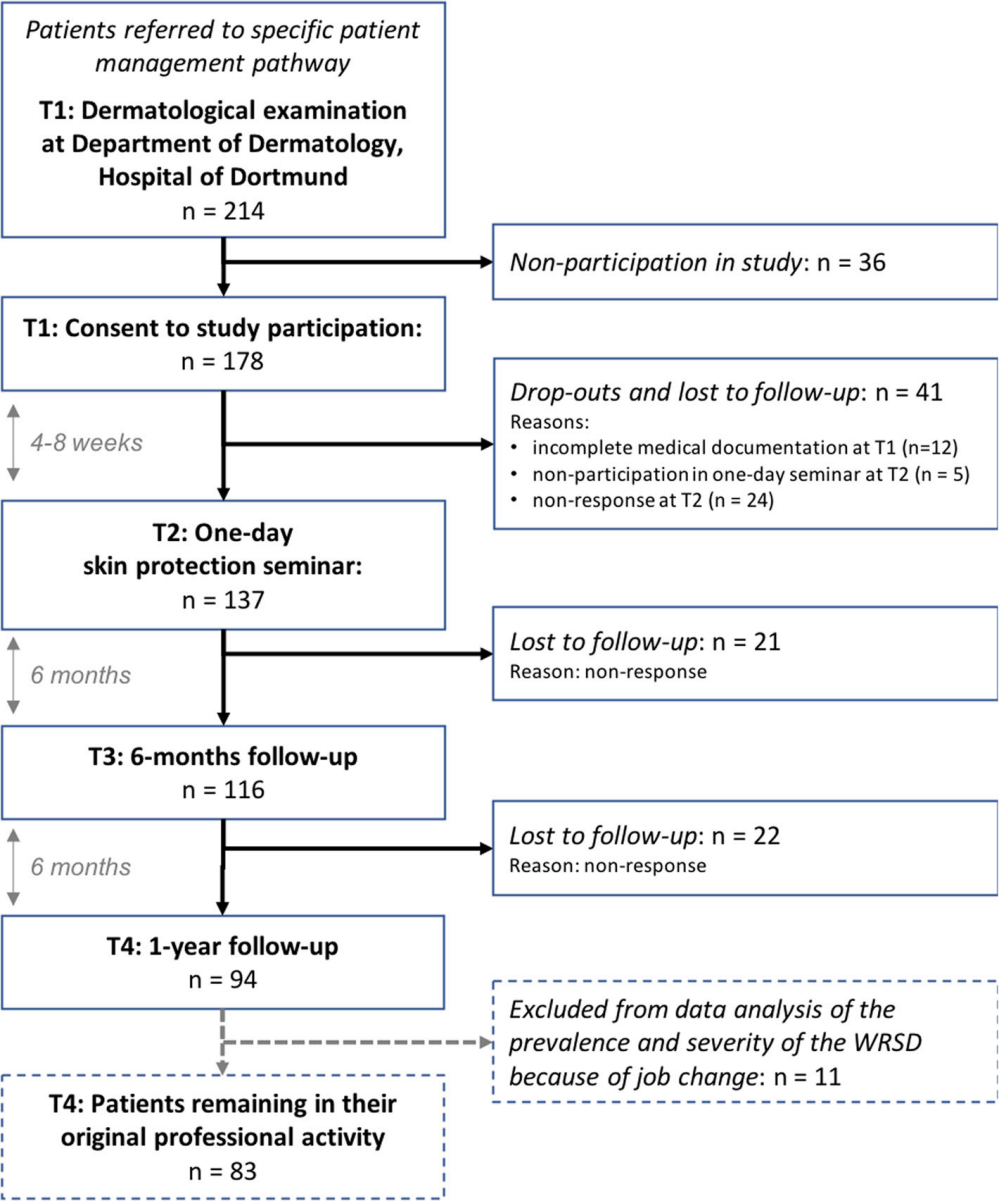
Flow chart and overview of the intervention and the study design (T1, T2, T3, T4)

Four to eight weeks after T1 (Fig. 1), an interdisciplinary, multiprofessional one-day skin protection seminar is conducted at the Hospital of Dortmund, which is near to the place of residence of most patients (T2). The seminar consists of both standardized group training and individual counseling. It emphasizes education and individual counseling and aims at empowering the patients’ coping with their WRSD, improving their self-management skills, increasing their motivation to perform an appropriate skin protection and skin care behavior. Furthermore, patients acquire disease-specific knowledge on the pathogenesis and prevention of WRSD. In addition, a choice of skin protection products is discussed and appropriate samples of skin protection products are provided. This is important since appropriate skin care and skin protection behavior can significantly influence the individual course of disease [13, 24, 25]. Table 1 shows the details of this one-day program.

**Table 1:** Description of the different interlinking modules of the interdisciplinary, multiprofessional one-day skin protection seminar (T2)

Module	Content	Method	Materials	Duration	Staff and institution
1	Welcoming and introduction to staff, participants, and program in order to create a trustful, open seminar atmosphere	Moderation of oral conversation (group)	Key questions for participants (e.g., occupation, risk factors)	15-20 min.	Health educationalist, University of Osnabrück
2	Legal basis concerning the statutory accident insurance, the procedure of patient care, differences and possible financial and social consequence of work-related and occupational diseases and compensation in order to increase the motivation to remain in work and to perform appropriate skin protection	Oral presentation, dialogue in case of individual questions (group)	Power point slides, script for notes	30 min.	Social security employee specialized in accident prevention, Social Accident Insurance Institution for the woodworking and metalworking industries, Dortmund
3	Skin protection seminar in order to gain disease-specific knowledge concerning the function and structure of the skin, anatomy of the stratum corneum, skin barrier function, external (e.g., cutting fluids, solvents) and internal (e.g., atopic diathesis) risk factors, pathogenesis of irritant and allergic contact dermatitis, and methods for skin protection, skin care and mild skin cleansing, to improve the disease management and to increase the motivation to perform skin protection behavior	Interactive, dialogue-oriented seminar (group)	Power point slides, flipcharts, hands-on skin protection experiments, pictures, metaphors, models (e.g., brick-and-mortar-model of the stratum corneum)	90-110 min.	Health educationalist, University of Osnabrück
4	Lunch break			45 min.	Cantina of the Hospital, Dortmund
5	Different types and usage of protective gloves, pictograms, hands-on showing of examples, explaining of appropriate and wrong usage (e.g., to avoid that gloves become contaminated on the inside)	Oral hands-on presentation, dialogue in case of individual questions (group)	Examples of various protective gloves to hand round to touch, feel and compare types, models, and pictograms, flipcharts	45 min.	Technical inspector, Social Accident Insurance Institution for the woodworking and metalworking industries, Dortmund
6	Circle with four stations:				
6a	Dermatological examination and counseling to assess the skin condition and the disease course, to recommend further diagnostics, to identify individual therapy options, and to answer individual questions	Individual patient counseling	Medical records (e.g., patch test results)	10-15 min.	Dermatologist, Department of Dermatology, Dortmund
6b	Review of the currently used skin protection products, if necessary: recommendation of alternative (optimized) protective gloves, skin creams, and/or mild skin cleanser for subsequent testing under real workplace conditions	Individual patient counseling	Report of workplace visit, different examples of protective gloves, creams, and cleansers	15 min.	Technical inspector, Social Accident Insurance Institution for the woodworking and metalworking industries, Dortmund
6c	Health educational counseling to answer individual questions, to explain the individual medical diagnosis with “simple words” (lay terms), to improve the individual skin protection behavior and to practice how to correctly apply cream	Individual patient counseling	Medical records, educational material to practice the correct application of cream and to identify “cream gaps”, other material (as individually required)	10 min.	Health educationalist, University of Osnabrück
6d	Counseling regarding subsequent steps and procedures (e.g., organizational inquiries, provision of skin products and gloves, information on future support by the social accident insurance)	Individual patient counseling	Documents and forms	10 min.	Social security employee specialized in accident prevention, Social Accident Insurance Institution for the woodworking and metalworking industries, Dortmund

Seven to ten of these one-day seminars are offered every year depending on the demand (number of WRSD cases annually reported to the social accident insurance). On average, they are held in small groups of 6–10 patients.

In addition, the disease severity and the course of disease between T1 and T2 is monitored at this seminar (T2) and the one-day seminar can serve as “guidepost” for further decisions on individual treatment needed, such as further consultations at the Department of Dermatology (Hospital of Dortmund), further diagnostics (e.g., patch testing, biopsy) or inpatient rehabilitation.

In summary, the prevention program consists of a behavior-oriented approach (e.g., the seminar to improve the individual skin protection behavior) and a structural approach (e.g., workplace visits, provision of skin protection, communication with the employer) offered in addition to the standard treatment at the local dermatologists.

### Study design and recruitment

We conducted an observational study with four measurement points (T1, T2, T3, T4, longitudinal pre/post-test design) in metal workers diagnosed with WRSD (Fig. 1).

At T1, recruitment was consecutively performed by the responsible dermatologist of the Department of Dermatology, Hospital of Dortmund. Since January 2013, every patient with a suspected WRSD who had been referred to this department has been asked for participation in this observational study. The participants gave informed written consent at baseline. They were informed about the fact that participation is voluntary and about the possibility to withdraw their consent at any time without any personal disadvantages. The study and the consent procedure were approved by the Ethics committee of the University of Osnabrück (Az.: 4/71043.5–1). Inclusion criteria for this observational study were a suspected WRSD, informed written consent, age of 18 or older, sufficient German language skills to handle written questionnaires, and an employment in a metalworking profession.

At T1, the participants filled out a standardized written questionnaire and the dermatologists assessed the skin condition and the atopy score. Four to eight weeks after T1, the participants took part in the multiprofessional one-day seminar (T2), filled out a written questionnaire at the end of the day, and the dermatologist assessed the skin condition for each participant. Six and twelve months after T2, the participants were followed up by the University of Osnabrück by postal written questionnaires (T3, T4) (Fig. 1). In case of non-response, two reminders were sent each at intervals of one month. The questionnaires at T1-T4 (Additional file 1) were identical in terms of all items to assess the outcomes presented in this paper. Items to evaluate the satisfaction with the one-day seminar (e.g., general conditions, structure, and comprehensibility) were only recorded at T2.

### Outcomes and instruments

The primary outcomes for this observational study are the number of patients who remain in their professional activity despite WRSD and the prevalence and severity of the WRSD. All outcomes have been assessed with standardized, written questionnaires either by dermatologists or patients.

The percentages of patients who remain in their professional activity were measured at T2, T3 and T4 with a closed question: “Do you still work in the same kind of professional activity as at the time of the first consultation at the Department of Dermatology in Dortmund (T2) / at the time of the skin protection seminar (T3/T4)?” (Additional file 1). If the response was “no”, the patients were asked if the change of professional activity was because of their skin disease or because of other reasons (e.g., other diseases, old-age pension).

At T1 and T2, the dermatologists assessed the severity of hand eczema with the Osnabrück Hand Eczema Severity Index (OHSI) on the basis of six morphological criteria (erythema, scaling, papules, vesicles, infiltration, fissures), with a possible score between 0 and 18, (higher values representing more severe diseases, cut-off point for “severe” hand eczema > 7 points) [26, 27]. They also assessed the diagnosis, the prevalence of WRSD at other parts of the body except from the hands, and the Erlanger atopy score (only at T1) for information on an atopic diathesis [28, 29] with higher values representing a higher likelihood of an atopic diathesis. As a usual part of the medical records (T1), and irrespective of study participation, the dermatologists rated the disease severity for all patients according to the criteria of the Bamberg Medical Bulletin (no, mild, medium, severe) [30], and with the occupational contact dermatitis disease severity index (ODDI) [31].

The participants rated their current disease severity at T1, T2, T3, and T4 with four self-assessment scales (for details of the rating systems see Table 3 and Additional file 1, applicable to all: the higher the rating the more severe the disease). One of these is based on the German school grades system consisting of six numerical grades (1: very good, 2: good, 3: satisfactory, 4: sufficient, 5: poor, 6: very poor). We chose this scale because we assumed that the majority of participants are familiar with the categories. Another instrument was a previously developed and validated photographic guide [32, 33]. As only self-reported data were available for T3 and T4, we calculated the correlation between the clinical gold standard (OHSI) at T1 and T2 and the different forms of self-assessment.

Socio-demographic characteristics, smoking habits (“Do you smoke?” yes/no), usage of steroids and other secondary outcomes were also assessed at T1-T4. All non-published questions that are relevant for the presented data are provided in Additional file 1.

### Data analysis

Data was stored and analyzed using IBM SPSS Statistics for Macintosh, Version 23.0 (IBM Corp., Armonk, NY, USA). For the primary outcomes, all data sets (T1-T4) have been checked for data entry mistakes to ensure quality of data input. For secondary outcomes, a sample of 45.2% of all data sets were checked for data entry mistakes (average error rate: 0.38% per data input). Descriptive statistics were calculated for all variables.

We have analyzed whether there are systematic differences between the study cohort and missing data (e.g. caused by non-participation in the study, lost to follow-up, Fig. 1). In case of missing values caused by participants omitting single questions, these are reported in the tables as differences missing to 100%.

Either Pearson’s chi-square test or Fisher’s exact test were used to analyze group differences of nominal variables (e.g. as part of dropout analyses). Fisher’s exact test was applied if at least one expected value under independence is lower than 5.

Parametric tests were used for statistical analysis of metric variables (e.g., as regards the OHSI and the atopy score). T-tests were applied for comparing the means of changes over time in dependent, paired samples and for comparing the means of independent subgroups at one point in time.

For ordinal variables, Wilcoxon’s non-parametric rank sum test was used to analyze differences in the mean ranks over time, and the Mann-Whitney-U-test was used for analyzing differences between two independent subgroups.

A repeated measures ANOVA with the within-subject factor, “self-reported disease severity” with four levels (time points T1, T2, T3, T4) was conducted to analyze changes in the self-assessed disease severity over time.

Pearson’s correlation coefficient and Spearman’s Rho were calculated to investigate the agreement between the clinical gold standard (OHSI) and the four different variables assessing the self-reported disease severity. Differences of correlations were examined by the Meng test [34]. For all tests, a significance level of 0.05 was chosen.

## Results

### Study cohort and drop-outs

Figure 1 shows a flow chart of the study cohort. Between January 2013 and April 2016, 214 consecutive patients with suspected WRSD were referred to the Department of Dermatology. From these patients, 178 (83.2%) agreed to participate in the observational study. Based on the medical records at T1, we did not find statistically significant differences between the study participants and the non-participants in terms of age, sex, atopy signs, diagnosis, positive patch test results and disease severity according to the ODDI and the Bamberg Medical Bulletin.

In the 6- and 12-months postal follow-ups, response rates of 83.7% (*n* = 149, T3) and 71.9% (*n* = 128, T4) were obtained. In the course of the study (T1-T4), drop-outs and lost to follow-up occurred because of different reasons as shown in Fig. 1. Finally, data sets for T1-T4 were available for 94 participants which, however, also contained some missing values in case of participants omitting single questions. This cohort was used for subsequent analyses.

We did not find significant differences at T1 between the study cohort (*n* = 94) and the drop-outs (*n* = 84) with regard to the outcomes presented in this paper as well as for other secondary outcomes (e.g., regarding disease specific knowledge, data not shown in this paper) except for one variable: there was a significantly higher percentage of patients diagnosed with irritant contact dermatitis in the study cohort (78.7%) compared to the drop-outs caused by lost to follow-up (57.5%) (*p* = 0.003, χ^2^ = 8.697, df = 1). However, no significant differences were found for other diagnoses (e.g., allergic contact dermatitis, psoriasis palmaris, or atopic hand eczema).

### Socio-demographic and work-related characteristics

The study cohort predominantly consists of men (92.6%, *n* = 87) and seven female participants (7.4%) with a mean age of 45.4 years (SD: 10.5, range: 18–62). Thirty-two patients (34.0%) reported to smoke.

At the baseline, all participants were employed and worked full-time as metal workers (e.g., cutting machine operators, machine fitters) or in appendant professions (e.g., automotive technicians). The most frequent vocational qualification was an apprenticeship (76.6%, *n* = 72), followed by unskilled workers (no vocational training, 9.6%, *n* = 9), master/technical school (8.5%, *n* = 8) and one patient each with higher education (1.1%) and an “other” vocational degree (1.1%).

### Remaining in work

The parameter ‘remaining in work’ is a main outcome as WRSD may finally lead to job loss. At T3 (six months) and T4 (twelve months), 94.7% (*n* = 89) and 88.3% (*n* = 83), respectively, still worked in the same professional activity as at T1.

At T3 and T4, two (2.1%) and four patients (4.3%) reported that they had given up their original professional activity mainly because of the skin disease. ‘Other reasons’ for not remaining in their professional activity were following further vocational training, insolvency, in-house transfer to another position due to restructuring of the business, dismissal or change of employer. From T1 to T4, no notable changes in the working time (full-time, part-time, unemployment) have been observed.

### Dermatological examination and atopy score

The prevalence and severity of the WRSD is the second primary outcome. As an improvement of the WRSD could also result from a job change being associated with reduced skin exposure to causal triggers, only patients remaining in their original professional activity at T4 were included in the analysis of this outcome (*n* = 83).

At T1, the mean OHSI score was 4.99 (SD: 2.64) and the mean atopy score was 6.9 (SD: 4.36) (Table 2). For about one-fifth of the patients (21.7%, *n* = 18) an atopic diathesis could be assumed, and for 18.1% (*n* = 15) it could be excluded. We found no significant correlation between the atopy score and the OHSI score (*r* = 0.217, *p* = 0.054, *n* = 79).

**Table 2:** Results of the dermatological examination at T1 and T2 (*n* = 83)

		T1	T2
OHSI score [total, for both hands]	mean [SD, range]	4.99 [2.64, 0–13]	3.90 [2.46, 0–11]
diagnosis [hands] (multiple answers possible)	irritant/cumulative subtoxic contact dermatitis [%, n]	80.7 [67]	89.2 [74]
	allergic contact dermatitis [%, n]	7.2 [6]	7.2 [6]
	atopic hand eczema [%, n]	12.0 [10]	9.6 [8]
	psoriasis palmaris [%, n]	13.3 [11]	10.8 [9]
	other, not classifiable [%, n]	2.4 [2]	3.6 [3]
atopy score	mean [SD, range]	6.9 [4.36, 0–20]	n. a.
	no atopic diathesis, 0–3 points [%, n]	18.1 [15]	n. a.
	atopic diathesis unlikely, 4–7 points [%, n]	41.0 [34]	n. a.
	atopic diathesis unclear, 8–9 points [%, n]	19.3 [16]	n. a.
	atopic diathesis, 10 or more points [%, n]	21.7 [18]	n. a.

*WRSD* work-related skin diseases, *T1* study enrollment, *T2* after the one-day program, *n* absolute number, *SD* standard deviation, *n. a*. not applicable/the atopy score was only assessed at T1

The vast majority of patients were diagnosed with irritant contact dermatitis (frequently used synonym in Germany: cumulative subtoxic contact dermatitis) of the hands at T1 (80.7%) (Table 2). In some cases, mixed diagnoses were determined (e.g. irritant contact dermatitis combined with atopic hand eczema). At T1, the dermatologists described in eleven patients (13.3%) that other parts of the body were also affected by WRSD, which mostly included the arms (*n* = 11), followed by legs (without feet, *n* = 5), feet (*n* = 4) and the truncus (n = 1).

From T1 to T2, the mean OHSI scores significantly decreased from T1 (mean: 4.99) to T2 (mean: 3.90) (*p* < 0.001, *t* = 4.24, *df* = 78, 95% *CI* [0.618, 1.711]) (Table 2).

Similar to Brans et al. [35], we analyzed the mean OHSI scores at T1 and T2 between smokers and non-smokers in a sub-cohort with complete data on the smoking status and the OHSI at T1 and T2 (*n* = 94, excluded: patient with psoriasis or change of reported smoking behavior between T1 and T2). We found a tendency for higher mean OHSI scores in the smokers (T1: 5.35, T2: 3.97) compared to the non-smokers (T1: 4.86, T2: 3.81), but the differences were not statistically significant (T1: *p* = 0.478, *t* = − 0.71, *df* = 92, 95% *CI* [− 1.884, 0.888], T2: *p* = 0.820, *t* = − 0.23, *df* = 92, 95% *CI* [− 1.537, 1.220]).

### Self-reported characteristics of the skin disease

At T1, the patients stated that they had already been suffering from the skin disease for a mean duration of 5.1 years (SD: 6.8, range: 0.5–32). Over the entire study period, the vast majority of patients were in current dermatological treatment due to their WRSD (Table 3). At T1, 16.9% (*n* = 14) of patients denied to have used steroids in the previous 12 months in contrast to 44.6% (*n* = 37) at T4. Thus, the percentage of patients who did not use steroids for 12 months more than doubled.

**Table 3:** Results of self-reported outcomes concerning the skin disease at T1, T2, T3 and T4 (*n* = 83)

		T1	T2	T3	T4
dermatological treatment due to WRSD [at present]	no [%, n]	3.6 [3]	n. a.	10.8 [9]	18.1 [15]
	yes [%, n]	94.0 [78]	n. a.	89.2 [74]	81.9 [68]
sick leave due to WRSD in the last 12 months	no [%, n]	60.2 [50]	n. a.	n. a.	n. a.
	yes [%, n]	38.6 [32]	n. a.	n. a.	n. a.
sick leave due to WRSD since T1	no [%, n]	n. a.	68.7 [57]	n. a.	n. a.
	yes [%, n]	n. a.	15.7 [13]	n. a.	n. a.
sick leave due to WRSD since T2	no [%, n]	n. a.	n. a.	91.6 [76]	86.7 [72]
	yes [%, n]	n. a.	n. a.	7.2 [6]	9.6 [8]
skin condition of the hands [“How do you assess the skin condition of your hands at the moment on a scale from 0 (no skin disorders) to 10 (severe skin disorders)?”, eleven-step numerical rating scale from 0 to 10]	mean [SD, range]	4.96 [2.31, 0–10]	4.57 [2.12, 1–10]	3.64 [2.19, 0–10]	3.71 [2.43, 0–9]
school grade for the skin condition of the hands [“With which school grade do you assess the skin condition of your hands at the moment?”, six-step numerical rating scale from 1 (very good) to 6 (unsatisfactory)]	mean [SD, range]	3.80 [1.12, 1–6]	3.55 [0.99, 1–5]	3.25 [1.08, 1–6]	3.28 [1.20, 1–6]
assessment of the statement	not at all [%, n]	2.4 [2]	7.2 [6]	12.0 [10]	15.7 [13]
“*I currently have skin symptoms*” [five-step Likert scale]	mild [%, n]	33.7 [28]	33.7 [28]	42.2 [35]	39.8 [33]
	moderate [%, n]	43.4 [36]	37.3 [31]	31.3 [26]	31.3 [26]
	strong [%, n]	15.7 [13]	8.4 [7]	6.0 [5]	12.0 [10]
	very strong [%, n]	2.4 [2]	2.4 [2]	2.4 [2]	–
photographic guide: worst hand eczema ever experienced	almost healed [%, n]	4.8 [4]	4.8 [4]	8.4 [7]	8.4 [7]
	mild [%, n]	14.5 [12]	18.1 [15]	22.9 [19]	24.1 [20]
	medium [%, n]	42.2 [35]	34.9 [29]	44.6 [37]	42.2 [35]
	severe [%, n]	30.1 [25]	22.9 [19]	18.1 [15]	16.9 [14]
photographic guide: average hand eczema in the last 12 months	almost healed [%, n]	6.0 [5]	2.4 [2]	14.5 [12]	20.5 [17]
	mild [%, n]	41.0 [34]	37.3 [31]	48.2 [40]	45.8 [38]
	medium [%, n]	38.6 [32]	36.1 [30]	26.5 [22]	24.1 [20]
	severe [%, n]	4.8 [4]	3.6 [3]	3.6 [3]	1.2 [1]
photographic guide: hand eczema at present [“If you look at your hands right now: Which group of pictures corresponds to your hand eczema? Please choose the more affected hand.” five-step Likert scale with each four pictures for four groups (almost healed to severe)]	no hand eczema [%, n]	3.6 [3]	3.6 [3]	12.0 [10]	10.8 [9]
	almost healed [%, n]	15.7 [13]	21.7 [18]	31.3 [26]	28.9 [24]
	mild [%, n]	41.0 [34]	41.0 [34]	43.4 [36]	36.1 [30]
	medium [%, n]	28.9 [24]	14.5 [12]	9.6 [8]	19.3 [16]
	severe [%, n]	3.6 [3]	1.2 [1]	2.4 [2]	2.4 [2]
changes of the skin disorders since T2 [“Did you skin disorder change since you have participated in the skin protection seminar?”]	no (=remained the same) [%, n]	n. a.	n. a.	38.6 [32]	31.3 [26]
	yes [%, n]	n. a.	n. a.	60.2 [50]	67.5 [56]
	I don’t know [%, n]	n. a.	n. a.	1.2 [1]	–
“If yes: How did your skin disorder change?” (T3: *n* = 50, T4: *n* = 56)	healed [%, n]	n. a.	n. a.	4.0 [2]	14.3 [8]
	strong improvement [%, n]	n. a.	n. a.	40.0 [20]	28.6 [16]
	slight improvement [%, n]	n. a.	n. a.	48.0 [24]	46.4 [26]
	slight worsening [%, n]	n. a.	n. a.	2.0 [1]	3.6 [2]
	strong worsening [%, n]	n. a.	n. a.	4.0 [2]	5.4 [3]
“Do you attribute this change to participating in the skin protection seminar?” (T3: *n* = 50, T4: *n* = 56)	yes [%, n]	n. a.	n. a.	40.0 [20]	51.8 [29]
	in parts [%, n]	n. a.	n. a.	54.0 [27]	28.6 [16]
	no [%, n]	n. a.	n. a.	6.0 [3]	17.9 [10]

*WRSD* work-related skin diseases, *T1* study enrollment, T*2* after the one-day program/several weeks after T1, T3: six months after T2, T4: twelve months after T2, n: absolute number, missing to 100%: missing values, n. a.: not applicable

At T4, 86.7% (*n* = 72) of the study participants denied sick leave due to the WRSD in the last twelve months as opposed to 60.2% (*n* = 50) at T1 (Table 3).

At T4, 67.5% (*n* = 56) we have observed changes in their skin condition since having attended the skin protection seminar; of these, 26 reported a “slight” and sixteen a “strong” improvement and eight patients a clearing of the disease (Table 3). Fifty-four attributed all or part of the improvement to the seminar.

All four variables assessing the self-reported disease severity (numerical rating scale, school grades, Likert scale, photographic guide, Table 3) indicate a pronounced improvement of the skin disease at the patients’ hands. For instance, 10.8% (*n* = 9) state to have no current hand eczema according to the photographic guide at T4 as opposed to 3.6% (*n* = 3) at T1. Hand eczema mostly improved between T2 (seminar) and T3 (6-months follow-up).

The results of the correlations between the clinical gold standard (OHSI) and the four forms of self-assessment at T1 and T2 are shown in Table 4. All correlations were statistically significant with highest correlation coefficients for the five-step Likert scale at T1 and T2. We chose this correlation (OHSI vs. five-step Likert scale) as standard and applied the Meng test [34] to compare this correlation with the other three correlations between the self-assessment scales and the OHSI. Applying the Meng test shows that the correlation for the five-step Likert scale did not perform significantly better than the others.

**Table 4:** Correlation between the clinical gold standard (OHSI) and self-assessment scales

	T1 (first consultation)	T2 (after one day program)
OHSI vs. eleven-step numerical rating scale from 0 to 10	r = 0.487^a^, *p* < 0.001, n = 77	r = 0.416^a^, *p* < 0.001, n = 70
OHSI vs. six-step numerical rating scale from 1 to 6 (school grades)	r = 0.477^a^, *p* < 0.001, *n* = 76	r = 0.533^a^, *p* < 0.001, *n* = 70
OHSI vs. five-step Likert scale (verbal)	r_S_ = 0.536^b^, *p* < 0.001, *n* = 77	r_S_ = 0.547^b^, *p* < 0.001, *n* = 74
OHSI vs. five-step Likert scale (photographic guide)	r_S_ = 0.415^b^, *p* < 0.001, *n* = 73	r_S_ = 0.543^b^, *p* < 0.001, *n* = 68

^a^Pearson’s correlation coefficient ^b^Spearman’s Rho

*OHSI* Osnabrück Hand Eczema Severity Index

A repeated measures ANOVA with the within-subject factor “self-reported disease severity” (eleven-step numerical rating scale from 0 to 10) [with four levels (time points T1, T2, T3, T4)] showed a significant main effect (Greenhouse-Geisser *F*(2.09, 138.22) = 21.59, *p* < 0.001). This result proves that the shown decrease in the self-assessed disease severity over time (Table 3; row “skin condition of the hands”) is significant.

At T4, three patients reported that they had participated in an inpatient rehabilitation program because of their WRSD [3, 36] since the skin protection seminar (T2). Compared to the other participants, these patients showed slightly but not significantly increased OHSI scores at T1 and T2.

### Disease control and satisfaction with the prevention program

Improving the patients’ self-perceived disease control were further aims of the program. Thus, we asked the patients for their agreement or rejection to the statement “I think I can handle my disease well in the future.” The proportion of patients who positively agreed to this statement increased after the skin protection seminar (T2) from T1 (47.1%) to T2 (68.8%), T3 (66.7%) and T4 (67.0%). Similar agreements were found for the statement “I have my skin disease under control.” (T1: 28.6%, T2: 43.8%, T3: 55.1%, T4: 61.1%).

At T2, we evaluated the satisfaction with the multidisciplinary one-day program, for instance in terms of seminar topics (e.g., importance of the topics, practical advices, comprehensibility), design and results of the seminar (e.g., program, atmosphere, better understanding of WRSD and risk factors), general conditions (e.g., organization, premises, catering) and the individual counseling rounds. Almost all items revealed a high level of approval and satisfaction between 81.9 and 100%. Nearly all participants (96.4%, T2) perceived their attendance in the one-day seminar as worthwhile. However, the evaluation indicates that more time could be spent on the exchange of experiences between the participants and on the topics “coping with stress” and “coping with itching”.

After one year (T4), 80.7–86.5% of the participants stated that they were satisfied with the protective gloves, skin creams and skin cleanser, which they currently use. Furthermore, 89.2% would recommend the seminar, 84.0% could put numerous tips received in the seminar into practice, and 83.0% evaluated their participation in the skin protection seminar as helpful. Several open comments indicated that “feeling informed” about their own skin disease, skin protection and medical treatment, as well as giving time for individual information and counseling, were very important and relevant outcomes of the prevention program as perceived by the patients.

## Discussion

In this year-long follow-up of a patient cohort, we examined the effects of a comprehensive outpatient interdisciplinary prevention program for metal workers with suspected WRSD. After one year, 88.3% (*n* = 83) of the participants remained in their original professional activity and four patients (4.3%) related a change of professional activity to their skin disease. The self-reported disease severity significantly improved one year after the skin protection seminar. The study also indicates an increased self-perceived disease control and an overall high satisfaction with the prevention program.

### Response-rates and drop-outs

We consider the response rates of 83.8% (T3) and 71.9% (T4) as satisfactory with regard to the follow-up period of one year. It is comparable to the response rates of intervention groups in previous follow-up studies in other professions [11–13, 15].

We exclusively included participants with data sets for all four measurements to the analysis. This led to a reduction of the analyzed study sample but to an improved comparability of data. We did not find significant differences between the study sample and the drop-outs caused by loss to follow-up after study enrollment, except for the variable “prevalence of irritant contact dermatitis” (ICD); in this regard there was a significantly higher percentage of patients diagnosed with ICD in the study cohort compared to the drop-outs. Since this exception was not found for other diagnoses (e.g. allergic contact dermatitis, psoriasis palmaris) we cannot think of any meaningful reason for this observation and consider this as an incidental finding. A selection bias cannot be excluded since the study sample (*n* = 94 and *n* = 83, Fig. 1) represents less than 50% of the entire patient cohort (*n* = 214). However, our (drop-out) analyses did not reveal further significant differences.

### Remaining in work

Apart from personal consequences for the individual worker (e.g. financial restrictions caused by unemployment), in times of demographic changes and a shortage of skilled workers also affecting the metalworking industries [37], it is of utmost importance for employers and the society to preserve the workforce of qualified employees.

One year after the skin protection seminar, 88.3% (*n* = 83) of the participants remained in their original professional activity. Four patients (4.3%) describe their skin disease as main reason for changing their profession. Compared to other follow-up studies of comparable outpatient prevention programs these results may be rated as very good although comparability is somewhat hampered by different follow-up times. Wilke et al. [12] found a proportion of 87.6 and 71.4% of workers in ‘wet work professions’ who remained in their former professional activity nine months and five years after an intervention, and 5.2 and 13.1% who related job loss to their WRSD. In a 6-year follow-up of geriatric nurses, 65.3 and 56.8% of an intervention and a control group (IG, CG), respectively, stayed in their jobs; 6.9% (IG) and 13.6% (CG) attributed job loss to WRSD [11]. In health care workers, Apfelbacher et al. [15] identified 8.7% who gave up working in their former professional activity due to their skin disease one year after a secondary individual prevention course and Soder et al. [17] reported the same for 9.2% (*n* = 12) in cleaning- and kitchen employees. Highest percentages were described in hairdressers of whom 12.8% of an intervention group and 27.3% of controls relate job loss to WRSD five years after an outpatient intervention [13].

To the best of our knowledge, no follow-up data have been published for the cohort of metal workers following secondary individual prevention in Germany. However, our data corroborate previous findings in other occupations and indicate that an interdisciplinary outpatient prevention program can have positive effects in metal workers in terms of the chance to remain in work despite WRSD.

In addition to results from intervention studies, the German Social Accident Insurance has data on the frequency of legally recognized occupational diseases no. 5101 which implies that the disease has forced the person to discontinue all activities that caused or could cause the disease [1]. In the year 2005 before the implementation of the hierarchical multi-step intervention procedure [‘Verfahren Haut’] by the German Social Accident Insurance [6–10], 879 cases of the occupational disease no. 5101 (“BK Nr. 5101”) were registered for all professions as compared to 515 cases in 2017. With respect to the group of “metal workers, mechanics and related professions” there were 107 cases in 2005 compared to 82 in 2017 (personal communication, S. Schneider, DGUV / German Social Accident Insurance, October 4, 2018). Thus, since intensified prevention programs have been established a decrease of the number of workers who had to give up working in their professional activity can be observed. However, this reduction cannot be solely attributed to the prevention program presented in this paper since other interventions such as inpatient rehabilitation programs have also been implemented at the same time.

### Dermatological examination

Our observation of irritant contact dermatitis being the most frequent medical diagnoses is supported by Skudlik et al. [36] who reported that 81.3% (*n* = 1357) of inpatient patients had an irritant component. We found only a few cases of WRSD in other body parts apart from the hands. This was expected since hands are the most frequent location for WRSD [23, 38] because they are usually exposed to irritants and allergens at workplace.

The OHSI (Osnabrück Hand Eczema Severity Index) has been used in previous studies [36, 39, 40]. In a workplace intervention study, Dulon et al. [39] reported a mean OHSI score of 3.5 (IG) and 3.2 (CG) points at baseline in geriatric nurses. Samardžic ´ et al. [40] found clinical skin symptoms in 40% of hairdressing apprentices with mean OHSI scores between 3.0 and 3.6. These scores were obtained in workers and not specifically in a patient cohort. Since our patient cohort was already under medical treatment, this explains the higher OHSI scores we observed (4.99, 3.90).

Skudlik et al. [36] described a mean OHSI of 6.3 for a large cohort of patients at admission to an intensified inpatient rehabilitation program that addresses patients suffering from severe WRSD. This explains the higher mean OHSI score at admission compared to our results of 4.99 at T1 for patients participating in an outpatient prevention program in case of milder forms of WRSD. For the inpatient cohort [36], the mean OHSI was 3.3 at one year [41] and 3.1 at three years after the rehabilitation program for patients who remained in the same professional field [3]. Our results of 3.90 at T2 as well as the significant decrease of the self-reported disease severity at T3 and T4 allow for a cautious forecast that our patient cohort will continue working in the same professional activity with a similar disease severity reported for the inpatient cohort even in the long term [3, 42].

The observation that some participants were free of hand eczema at T1 as well as a mild improvement between T1 and T2, can probably be attributed: to continuous therapy by the patient’s local dermatologist, to previous holiday leave or to an improved skin protection equipment and behavior resulting from the visit and counseling by an employee of the accident insurance at the workplace, which took place before T1.

Atopic skin diathesis has been described as potential risk factor for OSD [43–45] but we did not find a significant correlation between the OSHI and the atopy score. One possible explanation could be an increased awareness and skin care behavior particularly in “skin sensitive” persons [46].

We did not find a significantly higher OHSI score in smoking patients compared to non-smokers in contrast to the findings by Brans et al. [35]. Possible reasons could be that Brans et al. investigated a cohort of patients with severe WRSD as part of an inpatient rehabilitation program. Another reason might be methodological limitations of our study because a simple and single question (“Do you smoke?”) might not fully assess the smoking status (e.g., in terms of duration and quantity of cigarettes). Further, ex-smokers (about 43% of the German male population between 45 and 65 years) [47] were not identified in our cohort and excluded as done by Brans et al. In addition, social desirability might influence the answer to this question as smokers might fear possible disadvantages and thus deny their smoking behavior. The association between tobacco smoking and the prevalence and severity of WRSD in outpatient patients could be further and more comprehensively investigated in future studies.

### Self-reported disease severity

Whereas dermatological examinations are usually considered as gold standard for assessing the severity of hand eczema, it is often not possible for organizational reasons to perform them in follow-up studies. In such cases, self-reports are chosen to assess disease severity. In order to estimate the validity of self-reports at T3 and T4, we calculated the correlation between the OHSI and the four forms of self-assessment. Among these, the five-step verbal Likert scale correlated with the OHSI to the highest extent (T1: *r* = 0.536, *p* < 0.001; T2: *r* = 0.547, *p* < 0.001) followed by the other three variables, depending on the item and time of measurement (T1/T2, Table 4). According to Cohen’s conventions, r = 0.5 represents a large correlation [48]. However, it depends on the specific research context to rate a correlation as large, moderate or small. For individual diagnostic purposes, none of the four measures of self-reported disease severity is good enough to substitute the clinical gold standard (dermatological examination, OHSI) to monitor and diagnose the individual course of a disease. But the validity of the self-reports as part of a scientific study to compare or monitor group results over time is acceptable to conclude that the WRSD of our study has significantly improved from T1 to T4. This may be even more the case considering the fact that an interdisciplinary prevention program may even increase the subjective awareness of patients in terms of recognizing and interpreting even low-grade symptoms (e.g., dry skin) as clinical signs of hand eczema [12].

For future studies in this cohort (metal workers), only one or two items of self-assessment can be used in order to reduce the effort of the participants of processing the questionnaire and to increase the test economy. On the basis of our results, we prefer the easy-to-use five-step verbal Likert scale, which showed highest correlation. However, it is advisable to validate the assessment for self-reported disease severity in other cohorts (e.g., health-related or female-dominated occupations, inpatient cohorts, etc.) since the validity might vary according to the cohort and the individual perception of a disease.

According to the photographic guide (Table 3) nine patients (10.8%) stated that they were free of hand eczema at T4. In contrast, 72 reported that they still suffered from a mostly mild form of hand eczema. Therefore, it is a realistic goal of this and other comparable prevention programs to strive for ‘remaining at work and avoiding severe skin lesions’ instead of a complete and sustainable recovery. In terms of disease management and disease control, patients need to be informed about this prognosis and to be motivated regarding a continuous and appropriate skin protection behavior in the long-term.

### Approach of the prevention program

The prevention program presented in this paper systematically combines a behavior-oriented approach with a focus on the individual worker’s knowledge, attitudes and skin protection behavior in combination with mandatory elements of structural prevention. As described above, structural prevention comprises counseling and follow-up visits at the workplace by an employee of the prevention services of the responsible Social Accident Insurance (e.g., on-site identification of risks and possibilities for improved skin protection). This is important because the individual workplace situation in metalworking professions usually requires very specific demands regarding skin protection. These on-site elements at the workplace are structurally integrated in the pathways of individual patient care by the Social Accident Insurance Institution for the woodworking and metalworking industries. Other previously described interventions for outpatient individual prevention in Germany also incorporate health educational and dermatological interventions [11–16] but emphasize a behavior-oriented approach. Another special feature of the program is the multiprofessional one-day-seminar where patients and representatives of different disciplines (dermatology, health education, social accident insurance law, prevention services) get together in time and place for individual counseling and exchange.

The whole intervention approach is a typical “complex intervention” consisting of various interacting elements (e.g., different groups, complex behaviors, different outcomes) [49]. Answering the pivotal question of identifying “the active ingredients” of an intervention is a typical challenge in evaluation, since interventions are usually evaluated as a whole. Thus, future research in this field could systematically compare different outpatient prevention approaches with the same outcome sets in order to gain more insight into the question of “how much of which preventive approach (e.g., behavior or structural prevention) is most effective for my target group?” or “how much health education and individual counseling is needed?”

### Strengths and limitations

The observational study has some strengths and limitations. First of all, controls could not be taken for ethical and legal reasons as the prevention program forms part of the regular structures of patient care since 2007. Thus, the uncontrolled, longitudinal pre/post-test design as applied in comparable studies in Germany in the last 15–20 years, remains a feasible study design for this type of health services research [15–17, 23, 25, 50, 51]. However, an uncontrolled study cannot sufficiently prove the effectiveness of an intervention and the obvious methodological limitations regarding internal validity have to be considered for the interpretation of the results. Future studies could aim at further improving existing prevention programs and comparing the effects of existing prevention programs and enhanced programs (improved intervention). However, larger sample sizes will be needed since only small effects can be expected [52].

Another limitation is the reduction of the study cohort (*n* = 94 and *n* = 83 of 214 patients, Fig. 1) which might lead to a selection bias. However, (drop-out) analysis did not reveal significant differences between the different cohorts.

There are different scoring systems to clinically assess the severity of hand eczema, each with certain advantages and disadvantages [53]. We opted for the OHSI since it has been used in previous studies focusing on WRSD [3, 36, 39–42], and it is simple and easy to use in clinical settings to monitor disease severity [26, 27]. Previous follow-up studies of secondary individual prevention often exclusively relied on self-reports [11, 12, 15], which underpins a broader methodological strength of the present study.

In this follow-up, we have focused on distal primary outcomes that change and can only be measured a comparatively long time after the intervention. In contrast, more proximal outcomes (e.g., socio-cognitive variables and attitudes which might serve as determinants and prerequisites of skin protection behavior) are more directly affected by an intervention [51, 54], and changes can be observed immediately or shortly after an intervention. Analyzing these proximal outcomes allows more in-depth insights into effects of the prevention program and into behavior change processes of the patients. Future research should also focus on these proximal outcomes since results can be used to further improve and tailor the program to the specific needs of the target group.

There are some strengths of this study with regard to the use of the OHSI as a previously validated clinical instrument to assess disease severity [26, 27] and the validation of the self-reported disease severity. Future studies could investigate in more detail the validity of self-reported disease severity in different cohorts (e.g., possible gender-related differences, different professions or effects of patient education on the self-perceived disease severity). Another strength of this study is the inclusion of four follow-ups which allows the evaluation of changes over time.

## Conclusions

To the best of our knowledge, this is the first observational study that has investigated the effects of an interdisciplinary, multiprofessional skin protection program in metal workers with WRSD. Most metal workers were able to stay in their professional activity despite a continuing exposure to skin irritants and allergens. They nonetheless reported a significant improvement of their WRSD over time. Thus, our results corroborate previous findings in other professions that comprehensive, interdisciplinary outpatient prevention programs may positively influence the course of disease and increase the possibility to continue working in a “high-risk” profession.

## Additional file

Additional file 1: Questions and instruments**.** The file contains the non-published questions and instruments for T1-T4 that correspond to the data presented in the manuscript. It also contains references to instruments used in the study that have been developed before and published elsewhere. (DOCX 67 kb)

## Abbreviations

ACD: Allergic contact dermatitis
BGHM: Berufsgenossenschaft Holz und Metall
CG: Control group
ICD: Irritant contact dermatitis
IG: Intervention group
OHSI: Osnabrück Hand Eczema Severity Index
T: point in time (T1, T2, T3, T4)
WRSD: Work-related skin diseases
