# Supplementary material for: Interdisciplinary and multiprofessional outpatient secondary individual prevention of work-related skin diseases in the metalworking industry: 1-year follow-up of a patient cohort
Source: BMC Dermatol. 2018 Dec 12;18:12. doi: 10.1186/s12895-018-0080-2 (PMC6292163; doi:10.1186/s12895-018-0080-2)
Supplement: Supplementary file 1 — Questions and instruments. The file contains the non-published questions and instruments for T1-T4 that correspond to the data presented in the manuscript. It also contains references to instruments used in the study that have been developed before and published elsewhere. (DOCX 67 kb) [file 12895_2018_80_MOESM1_ESM.docx]

**Additional file 1: Questions and instruments**

This file contains the non-published questions and instruments for T1-T4 that correspond to the data presented in the manuscript. It also contains references to the instruments used in the study that have been developed before and published elsewhere. The non-published German questions used in the study have been translated to an English language version for the purpose of this publication.

[T1: Questions and instruments I](#_Toc531176182)

[T2: Questions and instruments IV](#_Toc531176183)

[T3: Questions and instruments VIII](#_Toc531176184)

[T4: Questions and instruments XI](#_Toc531176185)

# T1: Questions and instruments

## Personal details

**⯈ Your Gender:** 🗆 male 🗆 female

**⯈ Please state your profession or current professional activity:**

________________________________________________________________________

**⯈ How are you employed?**

🗆 self-employed 🗆 employed 🗆 unemployed

**⯈ Do you work full-time or part-time?**

🗆 full-time 🗆 part-time 🗆 unemployed

**⯈ What is your highest vocational qualification?**

🗆 no vocational training

🗆 apprenticeship

🗆 master/technical school

🗆 higher education

🗆 other vocational degree

**⯈ Do you smoke?** 🗆 no 🗆 yes 🗆 not stated

## Information about your skin disease

**⯈ How long have you been suffering from your skin disease?**

approx. _______ month(s) *or*  approx. _______ year(s)

**⯈ Have you been in dermatological treatment due to your work-related skin disease *in the last 12 months*?**

🗆 no 🗆 yes

**⯈ Are you *currently* in dermatological treatment due to your work-related skin disease?**

🗆 no 🗆 yes

**⯈ Have you used steroids for treating your skin disease in the last 12 months?**

🗆 no 🗆 yes, as cream 🗆 yes, as tablet

- 🡫

**If yes: how often?**  **If yes: how often?**

🗆 one-time / rare 🗆 one-time / rare

🗆 from time to time 🗆 from time to time

🗆 often 🗆 often

🗆 very often / almost non-stop 🗆 very often / almost non-stop

**⯈ Have you been unable to work *in the last 12 months* because of your work-related skin disease (“sick leave”)?**

🗆 no 🗆 yes

🡫

**If yes:** How many days? approx. ______ day(s)

**⯈ How do you assess the skin condition of your hands *at the moment* on a scale from 0 (no skin disorders) to 10 (severe skin disorders)?**

Please tick a number.

| 0 | 1 | 2 | 3 | 4 | 5 | 6 | 7 | 8 | 9 | 10 |
| --- | --- | --- | --- | --- | --- | --- | --- | --- | --- | --- |
| no  skin disorders |  |  |  |  |  |  |  |  |  | severe  skin disorders |

**⯈ With which school grade do you assess the skin condition of your hands *at the moment*?**

(1 = very good, 2 = good, 3 = satisfactory, 4 = sufficient, 5 = poor, 6 = very poor)

□ 1 □ 2 □ 3 □ 4 □ 5 □ 6

**⯈ “I currently have skin symptoms.”**

□ not at all □ mild □ moderate □ strong □ very strong

**⯈ “I think I can handle my disease well in the future.”**

□ exactly true □ very true □ moderately true □ hardly true □ not at all true

**⯈ “I have my skin disease under control.”**

□ exactly true □ very true □ moderately true □ hardly true □ not at all true

|  | exactly true | very true | moderately true | hardly true | not at all true |
| --- | --- | --- | --- | --- | --- |
| I am satisfied with the skin protection cream I use. | **□** | **□** | **□** | **□** | **□** |
| I am satisfied with the skin cleanser I use. | **□** | **□** | **□** | **□** | **□** |
| I am satisfied with the skin care cream I use. | **□** | **□** | **□** | **□** | **□** |
| I am satisfied with the protective gloves I use. | **□** | **□** | **□** | **□** | **□** |

## Photos to evaluate the disease severity

**⯈ Photographic guide, published by:**

Hald M, Veien NK, Laurberg G, Johansen JD. Severity of hand eczema assessed by patients and dermatologist using a photographic guide. Br J Dermatol. 2007;156(1):77-80.

Coenraads PJ, Van Der Walle H, Thestrup-Pedersen K, Ruzicka T, Dreno B, De La Loge C, Viala M, Querner S, Brown T, Zultak M. Construction and validation of a photographic guide for assessing severity of chronic hand dermatitis. Br J Dermatol. 2005;152(2):296-301.

## Dermatological examination

**⯈ Osnabrück Hand Eczema Severity Index (OHSI), published by:**

Dulon M, Skudlik C, Nübling M, John SM, Nienhaus A. Validity and responsiveness of the Osnabrück Hand Eczema Severity Index (OHSI): a methodological study. Br J Dermatol. 2009;160(1):137-42.

Skudlik C, Dulon M, Pohrt U, Appl KC, John SM. Osnabrueck hand eczema severity index – a study of the interobserver reliability of a scoring system assessing skin diseases of the hands. Contact Dermatitis. 2006;55(1):42-7.

**⯈ Erlanger atopy score, published by:**

Diepgen TL, Fartasch M, Hornstein OP. Kriterien zur Beurteilung der atopischen Hautdiathese. Dermatosen. 1991;39(3):79-83.

Diepgen TL, Sauerbrei W, Fartasch M. Development and validation of diagnostic scores for atopic dermatitis incorporating criteria of data quality and practical usefulness. J Clin Epidemiol. 1996;49(9):1031-8.

**⯈ Bamberg Medical Bulletin, published by:**

Diepgen TL, Bernhard-Klimt C, Blome O, Brandenburg S, Dienstbach D, Drexler H, Elsner P, Fartasch M, Frank KH, John SM, Kleesz P, Köllner A, Otten H, Pappai W, Römer W, Rogosky E, Sacher J, Skudlik C, Zagrodnik F. Bamberger Merkblatt: Begutachtungsempfehlungen für die Begutachtung von Haut- und Hautkrebserkrankungen. Dermatol Beruf Umwelt. 2008;56(4):132-50.

**⯈ Occupational contact Dermatitis Disease severity Index (ODDI), published by:**

Curr N, Dharmage S, Keegel T, Lee A, Saunders H, Nixon R. The validity and reliability of the occupational contact dermatitis disease severity index. Contact Dermatitis. 2008;59(3):157-64.

# T2: Questions and instruments

## Seminar evaluation

|  | exactly  true | very true | moderately true | hardly true | not at all  true |
| --- | --- | --- | --- | --- | --- |
| **Seminar topics:** | ☺☺ | ☺ | 😐 | ☹ | ☹☹ |
| The topics of the seminar were important for me. | **□** | **□** | **□** | **□** | **□** |
| I have received advices for practical use. | **□** | **□** | **□** | **□** | **□** |
| The contents were comprehensible. | **□** | **□** | **□** | **□** | **□** |
| My professional activity was taken into account. | **□** | **□** | **□** | **□** | **□** |
| I will put the suggestions into practice. | **□** | **□** | **□** | **□** | **□** |
|  |  |  |  |  |  |
|  |  |  |  |  |  |
|  | exactly  true | very true | moderately true | hardly true | not at all  true |
| **Seminar design:** | ☺☺ | ☺ | 😐 | ☹ | ☹☹ |
| I am satisfied with the course of the seminar | **□** | **□** | **□** | **□** | **□** |
| The exchange of experiences within the group was possible. | **□** | **□** | **□** | **□** | **□** |
| The atmosphere was pleasant. | **□** | **□** | **□** | **□** | **□** |
| My questions and experiences were taken into account. | **□** | **□** | **□** | **□** | **□** |
| Attending the seminar was worthwhile. | **□** | **□** | **□** | **□** | **□** |
| The presentations were appropriate and informative. | **□** | **□** | **□** | **□** | **□** |
|  |  |  |  |  |  |
|  |  |  |  |  |  |
|  | exactly  true | very true | moderately true | hardly true | not at all  true |
| **Seminar result:** | ☺☺ | ☺ | 😐 | ☹ | ☹☹ |
| I understand my skin disease better now. | **□** | **□** | **□** | **□** | **□** |
| I know my risk factors. | **□** | **□** | **□** | **□** | **□** |
| I can apply skin protection. | **□** | **□** | **□** | **□** | **□** |
| In the future I will be able to cope with my skin disease better. | **□** | **□** | **□** | **□** | **□** |
|  |  |  |  |  |  |
|  |  |  |  |  |  |
|  | exactly  true | very true | moderately true | hardly true | not at all  true |
| **General conditions:** | ☺☺ | ☺ | 😐 | ☹ | ☹☹ |
| I was satisfied with the organization before the seminar. | **□** | **□** | **□** | **□** | **□** |
| I was satisfied with the premises at the seminar location. | **□** | **□** | **□** | **□** | **□** |
| It is possible to arrange the seminar with my family obligations (e.g. care of children, relatives in need of care). | **□** | **□** | **□** | **□** | **□** |
| I was satisfied with the food. | **□** | **□** | **□** | **□** | **□** |

|  | exactly  true | | | very true | moderately true | | | hardly true | not at all  true | |  |
| --- | --- | --- | --- | --- | --- | --- | --- | --- | --- | --- | --- |
| **The following contents were comprehensible:** | ☺☺ | | | ☺ | 😐 | | | ☹ | ☹☹ | |  |
| Development of skin diseases | **□** | | | **□** | **□** | | | **□** | **□** | |  |
| Risk factors for the skin | **□** | | | **□** | **□** | | | **□** | **□** | |  |
| Treatment of skin diseases | **□** | | | **□** | **□** | | | **□** | **□** | |  |
| Application of skin protection, skin care and skin cleansing products | **□** | | | **□** | **□** | | | **□** | **□** | |  |
| Use of gloves | **□** | | | **□** | **□** | | | **□** | **□** | |  |
| Coping with stress and itching | **□** | | | **□** | **□** | | | **□** | **□** | |  |
|  |  | | |  |  | | |  |  | |  |
|  |  | | |  |  | | |  |  | |  |
|  | exactly  true | | | very true | moderately true | | | hardly true | not at all  true | |  |
| **Glove and skin product counseling:** | ☺☺ | | | ☺ | 😐 | | | ☹ | ☹☹ | |  |
| I am satisfied with the counseling I have received. | **□** | | | **□** | **□** | | | **□** | **□** | |  |
| There was sufficient time for questions. | **□** | | | **□** | **□** | | | **□** | **□** | |  |
| I consider the tested gloves and skin products suitable for my activities. | **□** | | | **□** | **□** | | | **□** | **□** | |  |
|  |  | | |  |  | | |  |  | |  |
|  |  | | |  |  | | |  |  | |  |
|  | exactly  true | | | very true | moderately true | | | hardly true | not at all  true | |  |
| **Medical counseling:** | ☺☺ | | | ☺ | 😐 | | | ☹ | ☹☹ | |  |
| I am satisfied with the counseling / examination I have received. | **□** | | | **□** | **□** | | | **□** | **□** | |  |
| There was sufficient time for questions. | **□** | | | **□** | **□** | | | **□** | **□** | |  |
|  |  | | |  |  | | |  |  | |  |
|  |  | | |  |  | | |  |  | |  |
|  | exactly  true | | | very true | moderately true | | | hardly true | not at all  true | |  |
| **Counseling in terms of legal aspects:** | ☺☺ | | | ☺ | 😐 | | | ☹ | ☹☹ | |  |
| I am satisfied with the counseling I have received. | **□** | | | **□** | **□** | | | **□** | **□** | |  |
| There was sufficient time for questions. | **□** | | | **□** | **□** | | | **□** | **□** | |  |
|  |  | | |  |  | | |  |  | |  |
|  |  | | |  |  | | |  |  | |  |
| **What did you particularly like?** | | | | | | | | | | | |
|  | | | | | | | | | | | |
|  | |  |  | | |  |  | | |  | |
| **What should we improve?** | | | | | | | | | | | |
|  | | | | | | | | | | | |

## Information about your professional activity

**⯈ Do you still work in the same kind of professional activity as at the time of the first consultation at the Department of Dermatology in Dortmund?**

🗆 no 🗆 yes

🡫

**If no: Why did you give up your professional activity?**

🗆 mainly because of my skin disease.

🗆 mainly for another reason (please tick):

🗆 other disease(s) 🗆 dismissal

🗆 advanced training 🗆 old age pension

🗆 parental leave

🗆 other reasons: ______________________________

## Information about your skin disease

**⯈ Have you been treated by your dermatologist because of your work-related skin disease since you first came to the Department of Dermatology at the Hospital of Dortmund?**

🗆 no 🗆 yes

**⯈ Have you used steroids for treating your skin disease since you first came to the Department of Dermatology at the Hospital of Dortmund?**

🗆 no 🗆 yes, as cream 🗆 yes, as tablet

- 🡫

**If yes: how often?**  **If yes: how often?**

🗆 one-time / rare 🗆 one-time / rare

🗆 from time to time 🗆 from time to time

🗆 often 🗆 often

🗆 very often / almost non-stop 🗆 very often / almost non-stop

**⯈ Have you been unable to work because of your work-related skin disease (“sick leave”) since you first came to the Department of Dermatology at the Hospital of Dortmund?**

🗆 no 🗆 yes

🡫

**If yes:** How many days? approx. ______ day(s)

**⯈ How do you assess the skin condition of your hands *at the moment* on a scale from 0 (no skin disorders) to 10 (severe skin disorders)?**

Please tick a number.

| 0 | 1 | 2 | 3 | 4 | 5 | 6 | 7 | 8 | 9 | 10 |
| --- | --- | --- | --- | --- | --- | --- | --- | --- | --- | --- |
| no  skin disorders |  |  |  |  |  |  |  |  |  | severe  skin disorders |

**⯈ With which school grade do you assess the skin condition of your hands *at the moment*?**

(1 = very good, 2 = good, 3 = satisfactory, 4 = sufficient, 5 = poor, 6 = very poor)

□ 1 □ 2 □ 3 □ 4 □ 5 □ 6

**⯈ “I currently have skin symptoms.”**

□ not at all □ mild □ moderate □ strong □ very strong

**⯈ “I think I can handle my disease well in the future.”**

□ exactly true □ very true □ moderately true □ hardly true □ not at all true

**⯈ “I have my skin disease under control.”**

□ exactly true □ very true □ moderately true □ hardly true □ not at all true

**⯈ Have you participated in an inpatient rehabilitation program because of your work-related skin disease since your first visit at the Department of Dermatology at the Hospital of Dortmund?**

🗆 no 🗆 yes

🡫

**If yes:** Where? ______________________________________________________

**⯈ Do you smoke?** 🗆 no 🗆 yes 🗆 not stated

|  | exactly true | very true | moderately true | hardly true | not at all true |
| --- | --- | --- | --- | --- | --- |
| I am satisfied with the skin protection cream I use. | **□** | **□** | **□** | **□** | **□** |
| I am satisfied with the skin cleanser I use. | **□** | **□** | **□** | **□** | **□** |
| I am satisfied with the skin care cream I use. | **□** | **□** | **□** | **□** | **□** |
| I am satisfied with the protective gloves I use. | **□** | **□** | **□** | **□** | **□** |

## Photos to evaluate the disease severity

**⯈ Photographic guide, published by:**

Hald M, Veien NK, Laurberg G, Johansen JD. Severity of hand eczema assessed by patients and dermatologist using a photographic guide. Br J Dermatol. 2007;156(1):77-80.

Coenraads PJ, Van Der Walle H, Thestrup-Pedersen K, Ruzicka T, Dreno B, De La Loge C, Viala M, Querner S, Brown T, Zultak M. Construction and validation of a photographic guide for assessing severity of chronic hand dermatitis. Br J Dermatol. 2005;152(2):296-301.

## Dermatological examination

**⯈ Osnabrück Hand Eczema Severity Index (OHSI), published by:**

Dulon M, Skudlik C, Nübling M, John SM, Nienhaus A. Validity and responsiveness of the Osnabrück Hand Eczema Severity Index (OHSI): a methodological study. Br J Dermatol. 2009;160(1):137-42.

Skudlik C, Dulon M, Pohrt U, Appl KC, John SM. Osnabrueck hand eczema severity index – a study of the interobserver reliability of a scoring system assessing skin diseases of the hands. Contact Dermatitis. 2006;55(1):42-7.

# T3: Questions and instruments

## Information about your professional activity and personal details

**⯈ Please state your profession or current professional activity:**

________________________________________________________________________

**⯈ Do you still work in the same kind of professional activity as at the time of the skin protection seminar?**

🗆 no 🗆 yes

🡫

**If no: Why did you give up your professional activity?**

🗆 mainly because of my skin disease.

🗆 mainly for another reason (please tick):

🗆 other disease(s) 🗆 dismissal

🗆 advanced training 🗆 old age pension

🗆 parental leave

🗆 other reasons: ______________________________

**⯈ How are you employed?**

🗆 self-employed 🗆 employed 🗆 unemployed

**⯈ Do you work full-time or part-time?**

🗆 full-time 🗆 part-time 🗆 unemployed

**⯈ Do you smoke?** 🗆 no 🗆 yes 🗆 not stated

## Information about your skin disease

**⯈ Have you been treated by your dermatologist because of your work-related skin disease since your participation in the skin protection seminar 6 months ago?**

🗆 no 🗆 yes

**⯈ Are you *currently* in dermatological treatment due to your work-related skin disease?**

🗆 no 🗆 yes

**⯈ Have you used steroids for treating your skin disease since your participation in the skin protection seminar 6 months ago?**

🗆 no 🗆 yes, as cream 🗆 yes, as tablet

- 🡫

**If yes: how often?**  **If yes: how often?**

🗆 one-time / rare 🗆 one-time / rare

🗆 from time to time 🗆 from time to time

🗆 often 🗆 often

🗆 very often / almost non-stop 🗆 very often / almost non-stop

**⯈ Have you been unable to work because of your work-related skin disease (“sick leave”) since your participation in the skin protection seminar 6 months ago?**

🗆 no 🗆 yes

🡫

**If yes:** How many days? approx. ______ day(s)

**⯈ Did you skin disorder change since you have participated in the skin protection seminar?**

🗆 no (=remained the same) 🗆 yes 🗆 I don’t know

🡫

⯈ **If yes: How did your skin disorder change?**

🗆 healed 🗆 slight worsening

🗆 strong improvement 🗆 strong worsening

🗆 slight improvement

**⯈ Do you attribute this change to participating in the skin protection seminar?**

🗆 yes 🗆 in parts 🗆 no

**⯈ How do you assess the skin condition of your hands *at the moment* on a scale from 0 (no skin disorders) to 10 (severe skin disorders)?**

Please tick a number.

| 0 | 1 | 2 | 3 | 4 | 5 | 6 | 7 | 8 | 9 | 10 |
| --- | --- | --- | --- | --- | --- | --- | --- | --- | --- | --- |
| no  skin disorders |  |  |  |  |  |  |  |  |  | severe  skin disorders |

**⯈ With which school grade do you assess the skin condition of your hands *at the moment*?**

(1 = very good, 2 = good, 3 = satisfactory, 4 = sufficient, 5 = poor, 6 = very poor)

□ 1 □ 2 □ 3 □ 4 □ 5 □ 6

**⯈ “I currently have skin symptoms.”**

□ not at all □ mild □ moderate □ strong □ very strong

**⯈ “I think I can handle my disease well in the future.”**

□ exactly true □ very true □ moderately true □ hardly true □ not at all true

**⯈ “I have my skin disease under control.”**

□ exactly true □ very true □ moderately true □ hardly true □ not at all true

**⯈ Have you participated in an inpatient rehabilitation program because of your work-related skin disease since your participation in the skin protection seminar?**

🗆 no 🗆 yes

🡫

**If yes:** Where? ______________________________________________________

|  | exactly true | very true | moderately true | hardly true | not at all true |
| --- | --- | --- | --- | --- | --- |
| I am satisfied with the skin protection cream I use. | **□** | **□** | **□** | **□** | **□** |
| I am satisfied with the skin cleanser I use. | **□** | **□** | **□** | **□** | **□** |
| I am satisfied with the skin care cream I use. | **□** | **□** | **□** | **□** | **□** |
| I am satisfied with the protective gloves I use. | **□** | **□** | **□** | **□** | **□** |

## Photos to evaluate the disease severity

**⯈ Photographic guide, published by:**

Hald M, Veien NK, Laurberg G, Johansen JD. Severity of hand eczema assessed by patients and dermatologist using a photographic guide. Br J Dermatol. 2007;156(1):77-80.

Coenraads PJ, Van Der Walle H, Thestrup-Pedersen K, Ruzicka T, Dreno B, De La Loge C, Viala M, Querner S, Brown T, Zultak M. Construction and validation of a photographic guide for assessing severity of chronic hand dermatitis. Br J Dermatol. 2005;152(2):296-301.

## Satisfaction with the skin protection seminar

|  | exactly true | very true | moderately true | hardly true | not at all true |
| --- | --- | --- | --- | --- | --- |
| I would recommend the skin protection seminar. | **□** | **□** | **□** | **□** | **□** |
| I can put numerous tips received in the seminar  into practice. | **□** | **□** | **□** | **□** | **□** |
| I could achieve my personal goals in terms of  seminar participation. | **□** | **□** | **□** | **□** | **□** |
| I can cope with my skin disease better since the skin protection seminar. | **□** | **□** | **□** | **□** | **□** |
| I personally find the participation in the seminar helpful. | **□** | **□** | **□** | **□** | **□** |

**⯈ *In retrospect: What did you particularly like about the skin protection seminar?***

|  |
| --- |

**⯈ *In retrospect: What should we improve?***

|  |
| --- |

# T4: Questions and instruments

## Information about your professional activity and personal details

**⯈ Please state your profession or current professional activity:**

________________________________________________________________________

**⯈ Do you still work in the same kind of professional activity as at the time of the skin protection seminar?**

🗆 no 🗆 yes

🡫

**If no: Why did you give up your professional activity?**

🗆 mainly because of my skin disease.

🗆 mainly for another reason (please tick):

🗆 other disease(s) 🗆 dismissal

🗆 advanced training 🗆 old age pension

🗆 parental leave

🗆 other reasons: ______________________________

**⯈ How are you employed?**

🗆 self-employed 🗆 employed 🗆 unemployed

**⯈ Do you work full-time or part-time?**

🗆 full-time 🗆 part-time 🗆 unemployed

**⯈ Do you smoke?** 🗆 no 🗆 yes 🗆 not stated

## Information about your skin disease

**⯈ Have you been treated by your dermatologist because of your work-related skin disease since your participation in the skin protection seminar 12 months ago?**

🗆 no 🗆 yes

**⯈ Are you *currently* in dermatological treatment due to your work-related skin disease?**

🗆 no 🗆 yes

**⯈ Have you used steroids for treating your skin disease since your participation in the skin protection seminar 12 months ago?**

🗆 no 🗆 yes, as cream 🗆 yes, as tablet

- 🡫

**If yes: how often?**  **If yes: how often?**

🗆 one-time / rare 🗆 one-time / rare

🗆 from time to time 🗆 from time to time

🗆 often 🗆 often

🗆 very often / almost non-stop 🗆 very often / almost non-stop

**⯈ Have you been unable to work because of your work-related skin disease (“sick leave”) since your participation in the skin protection seminar 12 months ago?**

🗆 no 🗆 yes

🡫

**If yes:** How many days? approx. ______ day(s)

**⯈ Did you skin disorder change since you have participated in the skin protection seminar?**

🗆 no (=remained the same) 🗆 yes 🗆 I don’t know

🡫

⯈ **If yes: How did your skin disorder change?**

🗆 healed 🗆 slight worsening

🗆 strong improvement 🗆 strong worsening

🗆 slight improvement

**⯈ Do you attribute this change to participating in the skin protection seminar?**

🗆 yes 🗆 in parts 🗆 no

**⯈ How do you assess the skin condition of your hands *at the moment* on a scale from 0 (no skin disorders) to 10 (severe skin disorders)?**

Please tick a number.

| 0 | 1 | 2 | 3 | 4 | 5 | 6 | 7 | 8 | 9 | 10 |
| --- | --- | --- | --- | --- | --- | --- | --- | --- | --- | --- |
| no  skin disorders |  |  |  |  |  |  |  |  |  | severe  skin disorders |

**⯈ With which school grade do you assess the skin condition of your hands *at the moment*?**

(1 = very good, 2 = good, 3 = satisfactory, 4 = sufficient, 5 = poor, 6 = very poor)

□ 1 □ 2 □ 3 □ 4 □ 5 □ 6

**⯈ “I currently have skin symptoms.”**

□ not at all □ mild □ moderate □ strong □ very strong

**⯈ “I think I can handle my disease well in the future.”**

□ exactly true □ very true □ moderately true □ hardly true □ not at all true

**⯈ “I have my skin disease under control.”**

□ exactly true □ very true □ moderately true □ hardly true □ not at all true

**⯈ Have you participated in an inpatient rehabilitation program because of your work-related skin disease since your participation in the skin protection seminar?**

🗆 no 🗆 yes

🡫

**If yes:** Where? ______________________________________________________

|  | exactly true | very true | moderately true | hardly true | not at all true |
| --- | --- | --- | --- | --- | --- |
| I am satisfied with the skin protection cream I use. | **□** | **□** | **□** | **□** | **□** |
| I am satisfied with the skin cleanser I use. | **□** | **□** | **□** | **□** | **□** |
| I am satisfied with the skin care cream I use. | **□** | **□** | **□** | **□** | **□** |
| I am satisfied with the protective gloves I use. | **□** | **□** | **□** | **□** | **□** |

## Photos to evaluate the disease severity

**⯈ Photographic guide, published by:**

Hald M, Veien NK, Laurberg G, Johansen JD. Severity of hand eczema assessed by patients and dermatologist using a photographic guide. Br J Dermatol. 2007;156(1):77-80.

Coenraads PJ, Van Der Walle H, Thestrup-Pedersen K, Ruzicka T, Dreno B, De La Loge C, Viala M, Querner S, Brown T, Zultak M. Construction and validation of a photographic guide for assessing severity of chronic hand dermatitis. Br J Dermatol. 2005;152(2):296-301.

## Satisfaction with the skin protection seminar

|  | exactly true | very true | moderately true | hardly true | not at all true |
| --- | --- | --- | --- | --- | --- |
| I would recommend the skin protection seminar. | **□** | **□** | **□** | **□** | **□** |
| I can put numerous tips received in the seminar  into practice. | **□** | **□** | **□** | **□** | **□** |
| I could achieve my personal goals in terms of  seminar participation. | **□** | **□** | **□** | **□** | **□** |
| I can cope with my skin disease better since the skin protection seminar. | **□** | **□** | **□** | **□** | **□** |
| I personally find the participation in the seminar helpful. | **□** | **□** | **□** | **□** | **□** |

**⯈ *In retrospect: What did you particularly like about the skin protection seminar?***

|  |
| --- |

**⯈ *In retrospect: What should we improve?***

|  |
| --- |
